# Diagnostic model for angiographic obstructive coronary artery disease combining CHG, DELC, and traditional risk factors: a bootstrap validation study

**DOI:** 10.3389/fendo.2026.1799141

**Published:** 2026-04-17

**Authors:** Wenxin Lin, Rui Gong, Mingliang Sun, Ruoling Guo, Yanying Yao, Bo Liu, Min Zhang, Xueyan Wang, Donglei Luo

**Affiliations:** 1 Graduate School, Chengde Medical University, Chengde, China; 2 Department of Cardiology, Chengde Central Hospital/Second Clinical College of Chengde Medical University, Chengde, China; 3 Clinical laboratory, Chengde Central Hospital/Second Clinical College of Chengde Medical University, Chengde, Hebei, China

**Keywords:** bootstraps approach, CHG, DELC, diagnostic model, obstructive coronary artery disease

## Abstract

**Objective:**

To construct and internally validate a diagnostic model for angiographic obstructive coronary artery disease (obstructive CAD) (defined as ≥50% stenosis on coronary angiography) incorporating the cholesterol, high-density lipoprotein, and glucose (CHG) index and diagonal earlobe crease (DELC) alongside other traditional risk factors.

**Methods:**

The study employed a cross-sectional design, involving a total of 1,645 patients, who were divided into two groups: those diagnosed with obstructive CAD (n = 1,298) and those without (n = 347). Independent risk factors were screened using least absolute shrinkage and selection operator (LASSO) regression and subsequently incorporated into a binary logistic regression model to construct the diagnostic model. The dose-response relationship between CHG and obstructive CAD risk was examined using restricted cubic spline analysis (RCS). The incremental diagnostic value was examined through DeLong’s test for area under the receiver operating characteristic curve (AUC) comparisons and through integrated discrimination improvement (IDI) and net reclassification improvement (NRI) for risk reclassification and discrimination improvement. Internal validation was performed using the Bootstrap method (B = 500 resamples). The model’s discriminative ability, calibration, and clinical utility were comprehensively assessed through a nomogram, AUC, calibration curve, decision curve analysis (DCA), and clinical impact curve (CIC).

**Results:**

Lasso regression ultimately identified six independent risk factors: male sex, hypertension, age, serum creatinine (Scr), CHG, and DELC. RCS revealed a linear positive correlation between the CHG index and obstructive CAD risk (P for nonlinear = 0.865). The constructed model yielded an apparent AUC of 0.692 (95% CI: 0.661–0.724) on the full dataset, with an optimistically corrected AUC of 0.683 (95% CI: 0.650–0.715) following internal validation via bootstrapping.

**Conclusion:**

CHG and DELC represent independent risk factors for obstructive CAD, with CHG levels exhibiting a linear relationship with obstructive CAD risk. The diagnostic model constructed based on these factors could assist in guiding subsequent diagnosis and treatment in high-risk populations.

## 1 Introduction

Angiographic obstructive coronary artery disease (obstructive CAD) is ranked among the leading causes of death and disability, presenting a critical public health issue (1). In China, the escalating prevalence of obstructive CAD has positioned it as a predominant cause of death across all demographics, concurrently exerting considerable economic pressure on societal resources and individuals (2). Therefore, early diagnosis of high-risk individuals and implementation of targeted prevention strategies are keys to reducing the incidence and mortality of obstructive CAD (3).

The pathological basis of obstructive CAD is atherosclerosis (AS), which is increasingly recognized not merely as a localized vascular disease, but rather as a systemic manifestation of endocrine and metabolic dysfunction (4). Insulin resistance (IR), as a key pathophysiological mechanism of metabolic abnormalities, plays a well-documented role in the development of obstructive CAD (5). This condition creates a complex endocrine-metabolic interplay, which not only impairs glucose homeostasis, leading to chronic hyperglycemia, but also disrupts systemic lipid metabolism (6). The cholesterol, high-density lipoprotein, and glucose (CHG) index quantitatively integrates atherogenic lipids with protective lipids. It may serve as a composite marker of underlying endocrine–metabolic dysregulation, and existing research has confirmed its diagnostic value for obstructive CAD risk events (7).

The diagonal earlobe crease (DELC) is an external manifestation reflecting endothelial dysfunction, microvascular lesions, or connective tissue degeneration, which shares a common pathological basis with AS (8–10). Several observational studies have suggested a possible link with obstructive CAD (11). As a simple and easily identifiable skin manifestation, it has a wide range of applications in obstructive CAD diagnosis (12).

Therefore, this study investigates the correlation between the CHG index and obstructive CAD risk, integrating it with DELC into a comprehensive diagnostic model to construct a more efficient risk stratification tool suitable for diverse clinical settings.

## 2 Materials and methods

### 2.1 Study population

**Figure 1:**
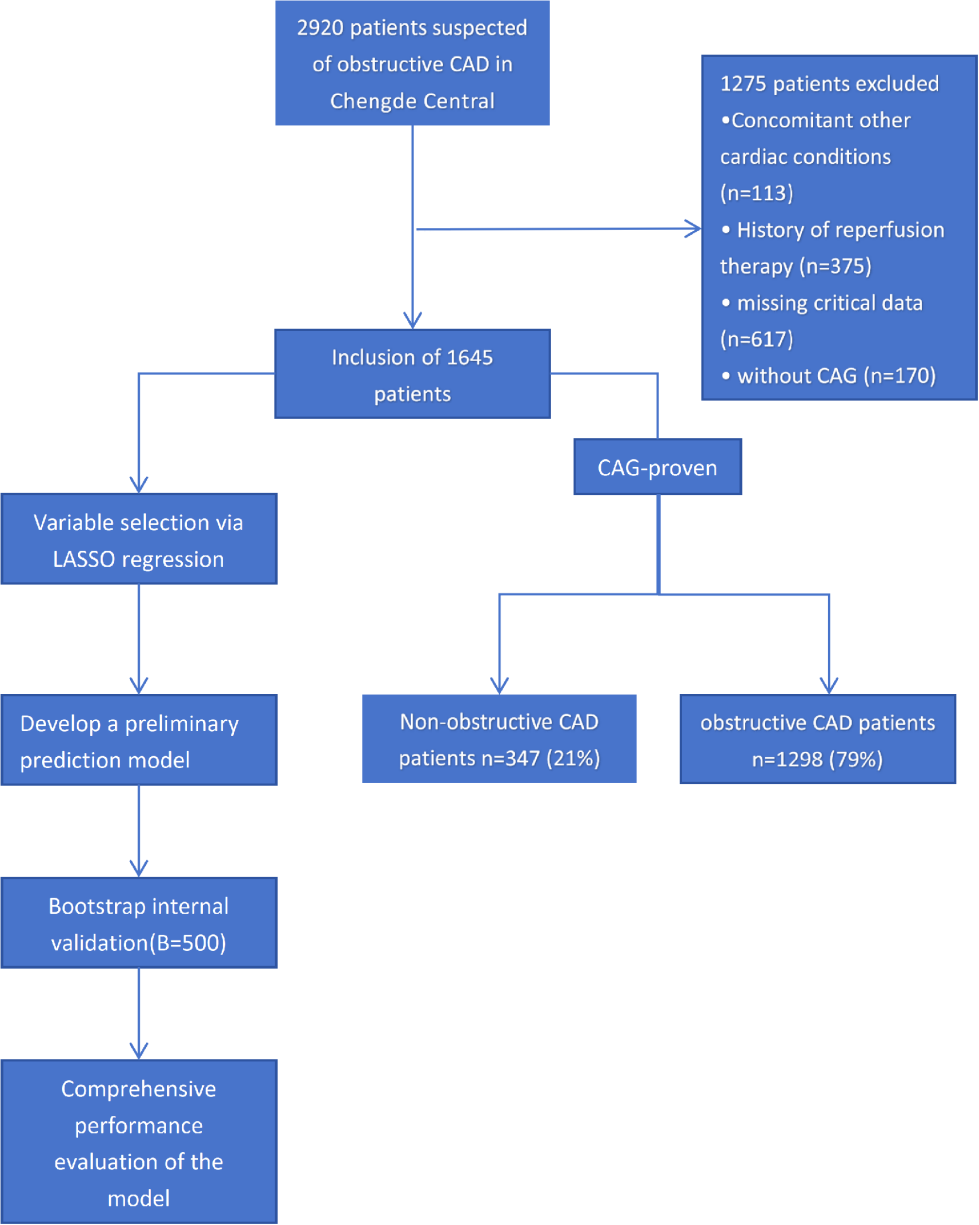
Study flowchart. Other cardiac conditions included: heart failure (New York Heart Association class ≥ II) and various valvular heart diseases. Key data were missing as follows: FBG (24%), TC (19%), and HDL-C (19%) were not recorded, and physical signs were not collected (38%). Obstructive CAD, obstructive coronary artery disease; CAG, coronary angiography.

Conducted between September 2021 and September 2025 at the Cardiovascular Department of Chengde Central Hospital, this prospective cross-sectional study comprised a cohort of 2,920 hospitalized patients manifesting chest pain with suspected obstructive CAD. Participants were selected based on the following criteria: (1) Chief complaint of chest pain or chest tightness; (2) Age≥18 years old; (3) Underwent coronary angiography (CAG). Exclusion criteria were as follows: (1) History of valvular heart disease and NYHA class≥II; (2) History of reperfusion therapy; (3) missing key data; (4) Having contraindications for CAG examination. In this prospective design, FBG, TC, HDL-C, and specific physical signs were critical variables linked to the dependent variable (obstructive CAD). As applying multiple imputation could risk artificially distorting the strength of their actual clinical associations, a strict complete-case analysis was maintained. This study has been approved by the Ethics Review Committee of Chengde Central Hospital. The Chinese clinical registration identifier for this trial is ChiCTR2000041499, which is accessible online at http://www.chictr.org.cn. The flowchart for this study is illustrated in **Figure 1**.

### 2.2 Variable definitions and data collection

#### 2.2.1 Demographic and clinical characteristics

Collection of patient baseline data: (1) Demographic characteristics: gender, age recorded as the exact calendar year at admission, BMI calculated from height and weight. (2) Medical history: hypertension, diabetes, smoking, and alcohol consumption. (3) Physical examination: systolic blood pressure (SBP), DELC. (4) Laboratory tests: initial admission panel including aspartate aminotransferase (AST), high-density lipoprotein cholesterol (HDL-C), fasting blood glucose (FBG), white blood cell count (WBC), Scr, low-density lipoprotein cholesterol (LDL-C), red blood cell count (RBC), alanine aminotransferase (ALT), hemoglobin (Hb), total cholesterol (TC), platelet count (PLT), thyroid-Stimulating hormone (TSH), triglyceride (TG), and echocardiogram. Specifically, Hypertension and diabetes were defined by prior physician-documented diagnosis or current use of antihypertensive or antidiabetic medication. Smoking was defined as a lifetime history of smoking (including current and former smokers). Alcohol consumption was defined as regular alcohol intake within the past year.

#### 2.2.2 CHG index

CHG index=Ln [(TC×FBG)/(2×HDL−C)]. (9)

The laboratory values for TC, FBG, and HDL-C were entered in mmol/L.

#### 2.2.3 DELC

DELC is a diagonal crease that extends across the earlobe from the tragus to the posterior auricular border. It is typically characterized by a depth of ≥1mm and a length covering at least one-third of the earlobe (8). All evaluating cardiovascular physicians undergo standardized clinical protocol training. The presence of DELC was independently assessed by two trained cardiovascular physicians. In instances of inter-rater diagnostic disagreement, a third senior cardiovascular specialist performed a final evaluation to guarantee objective morphological classification.

### 2.3 Obstructive CAD

All study participants underwent CAG. The examination is performed by an experienced cardiovascular interventionalist, routinely utilising the radial artery or femoral artery approach via the Judkins technique (13). All angiographic images were independently analysed by two interventional cardiologists unaware of the patients’ other clinical details. The diameter method was employed to assess the degree of coronary artery lumen stenosis. Where the two physicians disagreed on the severity of stenosis in any major vessel, a third, more senior interventional consultant physician reviewed the findings and made the final determination. According to the diagnostic thresholds established by the 2019 European Society of Cardiology guidelines (14), patients with angiographically proven stenosis ≥50% were assigned to the obstructive CAD group. Angiographic findings were recorded as binary categorical outcomes (<50% vs. ≥50%).

### 2.4 Statistical methods

This study employed SPSS 26.0, R software (version 4.5.0), and Prism 8.0 for statistical analysis and graphical representation. A two-sided p-value of less than 0.05 was defined as the threshold for statistical significance. For variables assessed for normality via the Kolmogorov-Smirnov test and quantile-quantile plots, those meeting the assumption of normality were reported as mean ± standard deviation, with between-group comparisons performed using Student’s t-tests; Variables not following a normal distribution are expressed as median (IQR). The Mann-Whitney U test was applied to compare non-normally distributed continuous variables. Descriptive statistics for categorical data are reported as case numbers (n) and percentages (%). Group differences for these variables were examined employing the chi-square test (c^2^). Principal component analysis (PCA) was performed on the selected variables to illustrate the multidimensional distribution of the study population. Risk factors were screened using least absolute shrinkage and selection operator (LASSO) regression and subsequently incorporated into a multivariable logistic regression model to develop a diagnostic model for obstructive CAD, ultimately presenting it as a nomogram. Internal validation was performed using the bootstrap method with 500 resampling iterations. In each resampling iteration, a bootstrap sample of the same size as the original dataset was drawn with replacement to develop the model. By comparing the original model with the bootstrap-corrected model using receiver operating characteristic (ROC) curves (AUC), decision curve analysis (DCA), clinical impact curves (CIC), and calibration curves to evaluate the model’s discriminatory power, calibration, and clinical utility. Comparison of AUCs between different models was performed using the DeLong test for paired ROC curves. Calculate the variance inflation factor (VIF) to examine multicollinearity among risk factors. Propensity score matching (PSM) was performed to evaluate the impact of age on other independent risk factors. Sample size is evaluated using the Events Per Variable (EPV) criterion. In our study, a total of 20 candidate predictors (including age, male, BMI, hypertension, diabetes, smoking, drinking, SBP, LVEF, WBC, RBC, Hb, PLT, TSH, ALT, Scr, TG, LDLC, DELC, and the CHG index), with 1,298 events of obstructive CAD, the EPV was 64.9, far exceeding the minimum requirement (≥10), ensuring sufficient statistical power.

## 3 Results

### 3.1 Demographic and clinical characteristics of the participants

The present study comprised a sample of 1,645 participants, including 1,298 individuals from the obstructive CAD group, and males accounted for 60.6%. The average age of the sample was 61.27 ± 9.39 years. The non-obstructive CAD group comprised 347 individuals, of whom 41.8% were male, with a mean age of 59.27 ± 9.09 years. Compared with the non-obstructive CAD group, the obstructive CAD group had a significantly higher percentage of males, hypertension, diabetes, smoking, and alcohol consumption, as well as a higher proportion of patients with DELC. Patients in the obstructive CAD group also demonstrated higher levels of age, SBP, WBC, Scr, FBG, RBC, TG, Hb, and CHG index than those in the non-obstructive CAD group. Conversely, their left ventricular ejection fraction (LVEF) and HDL-C levels were more depressed than those in the non-obstructive CAD group. All intergroup comparisons demonstrated statistically significant differences (*P* < 0.05). No statistically significant differences were observed between the two groups for the remaining indicators (*P* > 0.05). (**Table 1**).

**Table 1:** Baseline characteristics of the obstructive CAD and non-obstructive CAD group.

Variables	Obstructive CAD (n=1298)	Non-obstructive CAD (n=347)	*t/c^2^/z*	*P* value
Age(years)	61.27 ± 9.39	59.27 ± 9.09	-3.536	< 0.001
Male	787 (60.6%)	145 (41.8%)	39.597	< 0.001
BMI (kg/m^2^)	25.53 ± 3.52	25.55 ± 4.08	0.057	0.955
SBP (mmHg)	138.83 ± 19.81	135.36 ± 20.60	-2.870	0.004
Hypertension	846 (65.2%)	189 (54.5%)	13.462	< 0.001
Diabetes	374 (28.8%)	62 (17.9%)	16.841	< 0.001
Smoking	617 (47.5%)	115 (33.1%)	22.968	< 0.001
Drinking	439 (33.8%)	95 (27.4%)	5.186	0.023
LVEF (%)	58.00 (5.00)	60.00 (5.00)	-2.598	0.009
WBC (×10^9^)	6.30 (2.30)	6.00 (2.10)	-3.280	0.001
RBC (×10^12^/L)	4.62 ± 0.51	4.56 ± 0.49	-2.038	0.042
Hb (g/L)	142.69 ± 15.43	140.45 ± 14.72	-2.427	0.015
PLT (×109/L)	211.70 ± 54.67	215.27 ± 56.98	1.071	0.284
TSH (mIU/L)	2.37 (2.33)	2.43 (1.92)	-0.042	0.967
ALT (U/L)	21.00 (15.00)	20.00 (14.00)	-0.690	0.491
Scr (μmol/L)	69.07 ± 22.52	63.24 ± 15.88	-4.528	< 0.001
FBG (mmol/L)	5.50 (1.80)	5.30 (1.20)	-3.740	< 0.001
TG (mmol/L)	1.59 (1.24)	1.43 (0.99)	-3.148	0.002
TC (mmol/L)	4.33 ± 1.10	4.25 ± 1.04	-1.254	0.210
HDL-C (mmol/L)	1.12 ± 0.28	1.22 ± 0.33	5.167	< 0.001
LDL-C (mmol/L)	2.25 (1.08)	2.18 (1.08)	-1.761	0.078
CHG	5.34 ± 0.45	5.16 ± 0.40	-6.997	< 0.001
DELC	984 (75.8%)	220 (63.4%)	21.485	< 0.001

BMI, body mass index; SBP, systolic blood pressure; LVEF, left ventricular ejection fraction; WBC, white blood cell count; RBC, red blood cell count; Hb, hemoglobin; PLT, platelet count; TSH, thyroid-stimulating hormone; ALT, alanine aminotransferase; Scr, serum creatinine; FBG, fasting blood glucose; TG, triglyceride; TC:total cholesterol; HDL-C, high-density lipoprotein cholesterol; LDL-C, low-density lipoprotein cholesterol; CHG, cholesterol, high-density lipoprotein, and glucose; DELC, diagonal earlobe crease.

### 3.2 PCA

To visualize the multidimensional distribution of the study population and assess the separation between patients with angiographically diagnosed obstructive CAD and those without, PCA was conducted on the selected variables. The PCA revealed substantial overlap between the obstructive CAD and non-obstructive CAD groups. The first and second principal components explained 17.0% and 10.7% of the total variance, respectively, accounting for a cumulative 27.7%. Notably, the CHG index contributed significantly to the first dimension (Dim1) (**Supplementary Figure S3**). This observed multidimensional overlap further underscores the necessity of employing logistic regression models to achieve adequate diagnostic discrimination.

### 3.3 LASSO-based feature selection and logistic regression

Perform logistic regression analysis with obstructive CAD status (0=no, 1=yes) as the dependent variable. Using LASSO regression analysis with 10-fold cross-validation, six variables were selected at the λ_1se_ = 0.03104, effectively reducing the potential for model overfitting. The subsequent multivariable logistic regression analysis demonstrated that all six factors were independent predictors of obstructive CAD. Notably, the CHG index emerged as a powerful metabolic predictor (OR = 2.705, 95% CI: 1.984–3.689, P < 0.001). Other significant contributors included male sex (OR = 1.951, P < 0.001), age (OR = 1.025, P = 0.001), hypertension (OR = 1.430, P = 0.006), and DELC (OR = 1.405, 95% CI: 1.056–1.867, P = 0.019) (**Table 2**). The final diagnostic equation was constructed as follows:

**Table 2:** Multivariable logistic regression analysis of factors associated with obstructive CAD.

Variable	B	OR (95% CI)	P value
DELC	0.340	1.405 (1.056,1.867)	0.019
Age	0.025	1.025 (1.010,1.041)	<0.001
Male	0.668	1.951 (1.455,2.615)	<0.001
Hypertension	0.358	1.430 (1.108,1.846)	0.006
Scr	0.014	1.014 (1.004,1.024)	0.005
CHG	0.995	2.705 (1.984,3.698)	<0.001
Constant	-7.122	–	< 0.001

OR, odds ratio; CI, confidence interval; CHG, cholesterol, high-density lipoprotein, and glucose; DELC, diagonal earlobe crease; Scr, serum creatinine.

Logit(P)= −7.122 + 0.668×Male + 0.358×Hypertension + 0.025×Age + 0.014×Scr + 0.995×CHG + 0.340×DELC.

where *P* represents the individual predicted risk of obstructive CAD, and categorical variables (Male, Hypertension, DELC) are coded as 1 for presence and 0 for absence. Age and Scr are continuous variables, expressed in their original units of measurement.

**Figure 2:**
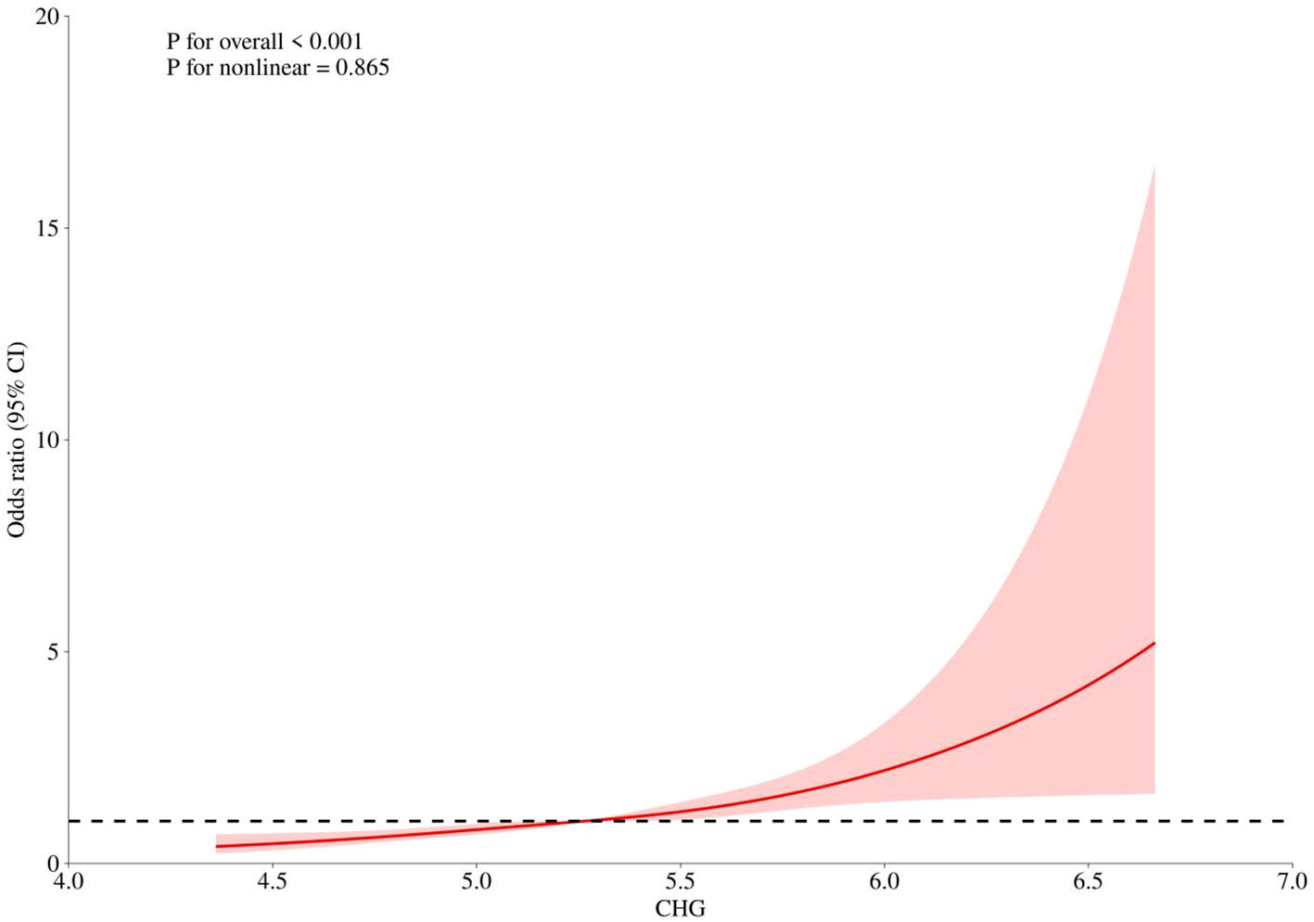
Nomogram of the diagnostic model for obstructive CAD constructed based on the CHG index, hypertension, age, DELC, Scr, and male sex. The nomogram is based on the apparent model without shrinkage. Predicted probabilities should be interpreted within the context of the study population. CHG, cholesterol, high-density lipoprotein, and glucose; DELC, diagonal earlobe crease; Scr, serum creatinine.

Calculating variance inflation factors (VIF) to test for multicollinearity among independent variables revealed that all VIF values were < 2, indicating extremely low linear correlation between variables. A diagnostic model was constructed based on the six independent risk factors identified above and was graphically represented by a nomogram. For example, a 55-year-old male patient with no prior history of hypertension presented with a positive DELC on physical examination. Laboratory tests revealed a Scr level of 75 μmol/L and a CHG index of 5.2. His total score was 122 points. Projecting this score vertically onto the risk axis indicated a corresponding obstructive CAD risk of 81.7%. (**Figure 2**).

### 3.4 Dose-response relationship between CHG and obstructive CAD risk

**Figure 3:**
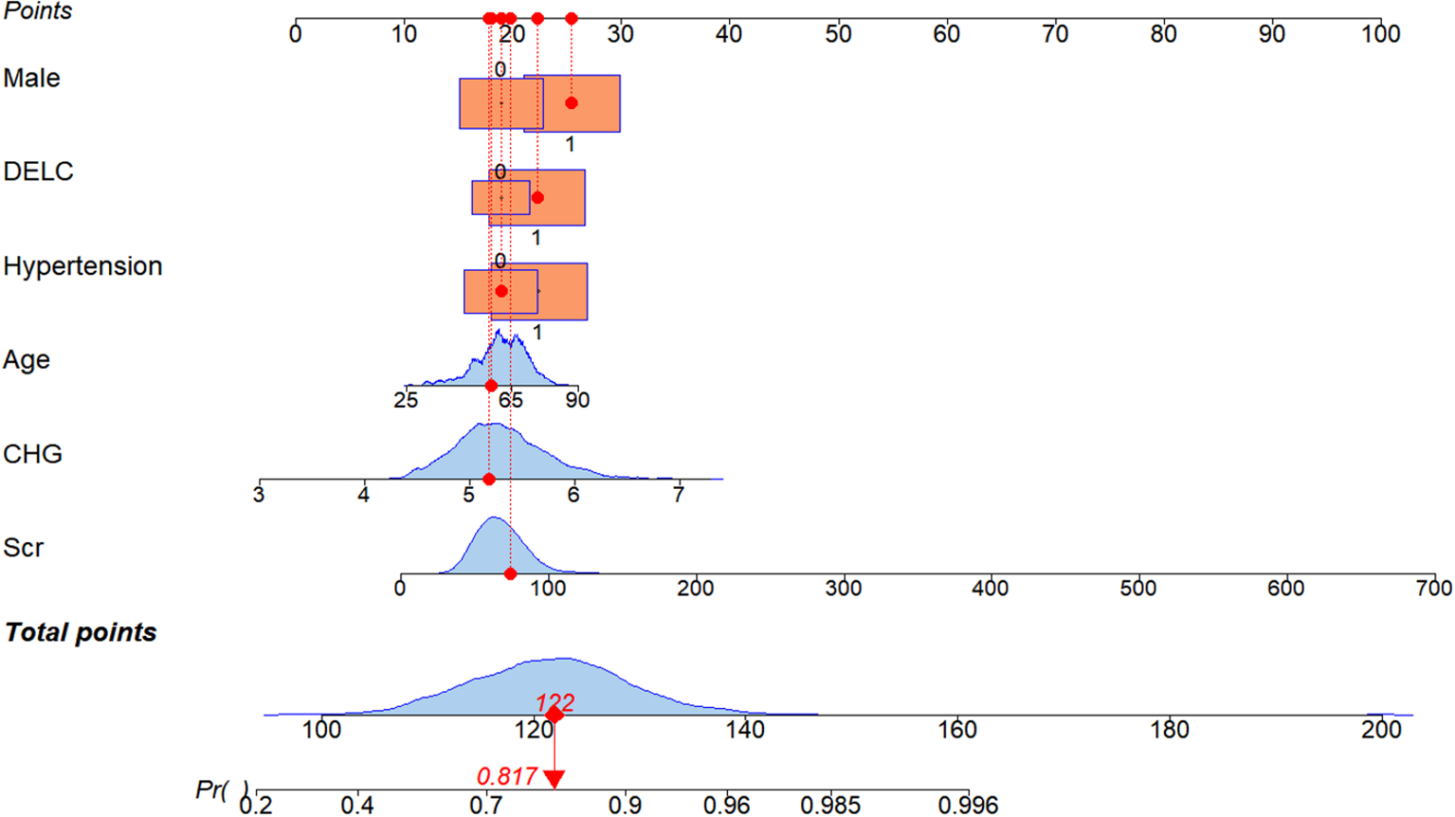
RCS of the association between CHG and the risk of obstructive CAD. RCS knots were placed at the 5th, 35th, 65th, and 95th percentiles of the CHG index, with the median as the reference. OR, odds ratio; CI, confidence interval; CHG, cholesterol, high-density lipoprotein, and glucose.

RCS regression was conducted using four knots positioned at the 5th, 35th, 65th, and 95th percentiles of CHG. The median CHG value served as the reference. Adjustments were made for age, sex, hypertension, and serum creatinine. Following adjustment for potential confounders, the RCS analysis revealed a statistically significant association between CHG levels and obstructive CAD risk (*P* for overall < 0.001), with CHG levels exhibiting a linear relationship with obstructive CAD risk (*P* for nonlinear = 0.865). This indicates that higher CHG levels correlate with increased obstructive CAD risk (**Figure 3**). It is noteworthy that the confidence intervals for the extreme high values of the CHG index were notably wide. Therefore, the dose-response relationship between extremely high CHG levels and obstructive CAD should be interpreted with caution.

### 3.5 Incremental value of CHG and DELC

We constructed four nested models to evaluate the incremental value of DELC and CHG.

Model 1: Age +Male + Hypertension +Scr;.
Model 2: Age +Male + Hypertension +Scr + CHG.
Model 3: Age +Male + Hypertension +Scr + CHG + DELC.
Model 4: Age +Male + Hypertension +Scr + DELC + FBG + TC + HDL-C.

As indicated by the results, the AUC values were 0.652 (95% CI: 0.619–0.685) for Model 1, 0.690 (95% CI: 0.658–0.721) for Model 2, 0.692 (95% CI: 0.661–0.724) for Model 3, and 0.694 (95% CI: 0.663–0.726) for Model 4.

DeLong’s test revealed that the inclusion of the CHG index significantly improved the discriminative performance of the model compared to the Model 1 (*P* < 0.001). After further incorporating DELC, the AUC increased to 0.692, which was significantly higher than that of Model 1 (*P* < 0.001), but showed no statistically significant difference compared to Model 2 (*P* = 0.490).

To evaluate the impact of the CHG index on the diagnostic model compared with its three individual components, Model 3 and Model 4 were compared. DeLong’s test revealed no statistically significant difference in discriminative ability between the two models (*P* = 0.331). The categorical NRI was -0.0086 (95% CI: -0.0186 - 0.0013, *P* = 0.089), the continuous NRI was -0.0528 (95% CI: -0.1711 - 0.0655, *P* = 0.381), and the IDI was -0.0020 (95% CI: -0.0046 - 0.0006, *P* = 0.136). The non-significant difference suggests that the CHG index preserves diagnostic accuracy while consolidating the three separate parameters into a unified composite indicator.

### 3.6 Validation of the model’s performance

#### 3.6.1 Model discrimination

**Figure 4:**
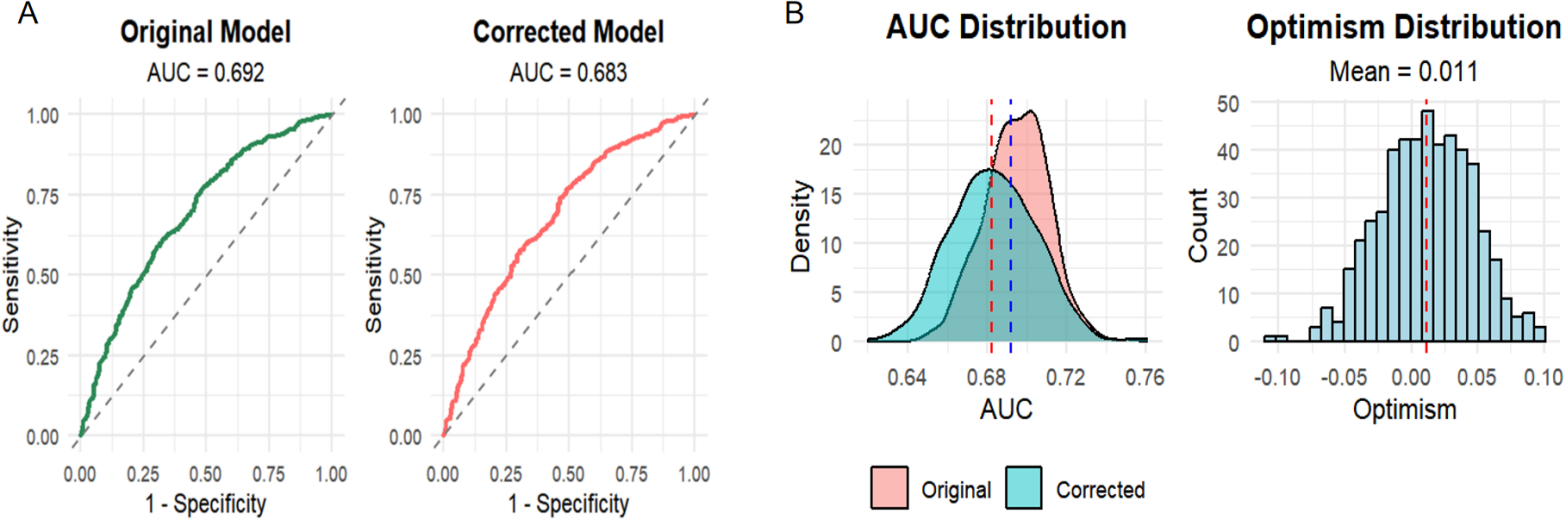
ROC curve analysis of the original and corrected diagnostic model for obstructive CAD. **(A)** ROC curve analysis of the original model. **(B)** ROC curve analysis of the corrected diagnostic model. The optimism in model performance was estimated as follows: a model was developed in a bootstrap sample and its apparent performance was assessed in that same sample; the model was then tested in the original full dataset. The difference between these two performance estimates was recorded as the optimism. AUC, area under the receiver operating characteristic curve.

The model achieved an AUC of 0.692 (95% CI: 0.661–0.724) on the original dataset. Following 500-sample bootstrap internal validation, the optimistically corrected AUC was 0.683 (95% CI: 0.650–0.715), indicating stable model performance with moderate discriminative capability. The mean optimism value of 0.011 suggests low overfitting and favourable generalisation ability (**Figure 4**). Although the λ_1se_ rule was employed to improve the stability of the selected variables, variable selection was performed only once on the full dataset and not repeated within each bootstrap resample. Consequently, the optimism-corrected estimates may be underestimated as the uncertainty inherent in the variable selection process was not fully accounted for. The degree of optimism correction provided should be interpreted with caution.

#### 3.6.2 Model calibration

The model showed acceptable calibration after 500 bootstrap resamples, with its estimated risks showing close approximation to the observed event rates. The calibration curve of the original model demonstrated good agreement with the ideal diagonal line. The calibration slope was 0.967, and the calibration-in-the-large (intercept) was 0.036, indicating good agreement between predicted and observed probabilities. The Brier score was 0.154, suggesting acceptable overall model performance. These results indicate that the nomogram is well-calibrated and maintains robust performance after adjusting for potential overfitting.

#### 3.6.3 Clinical utility

**Figure 5:**
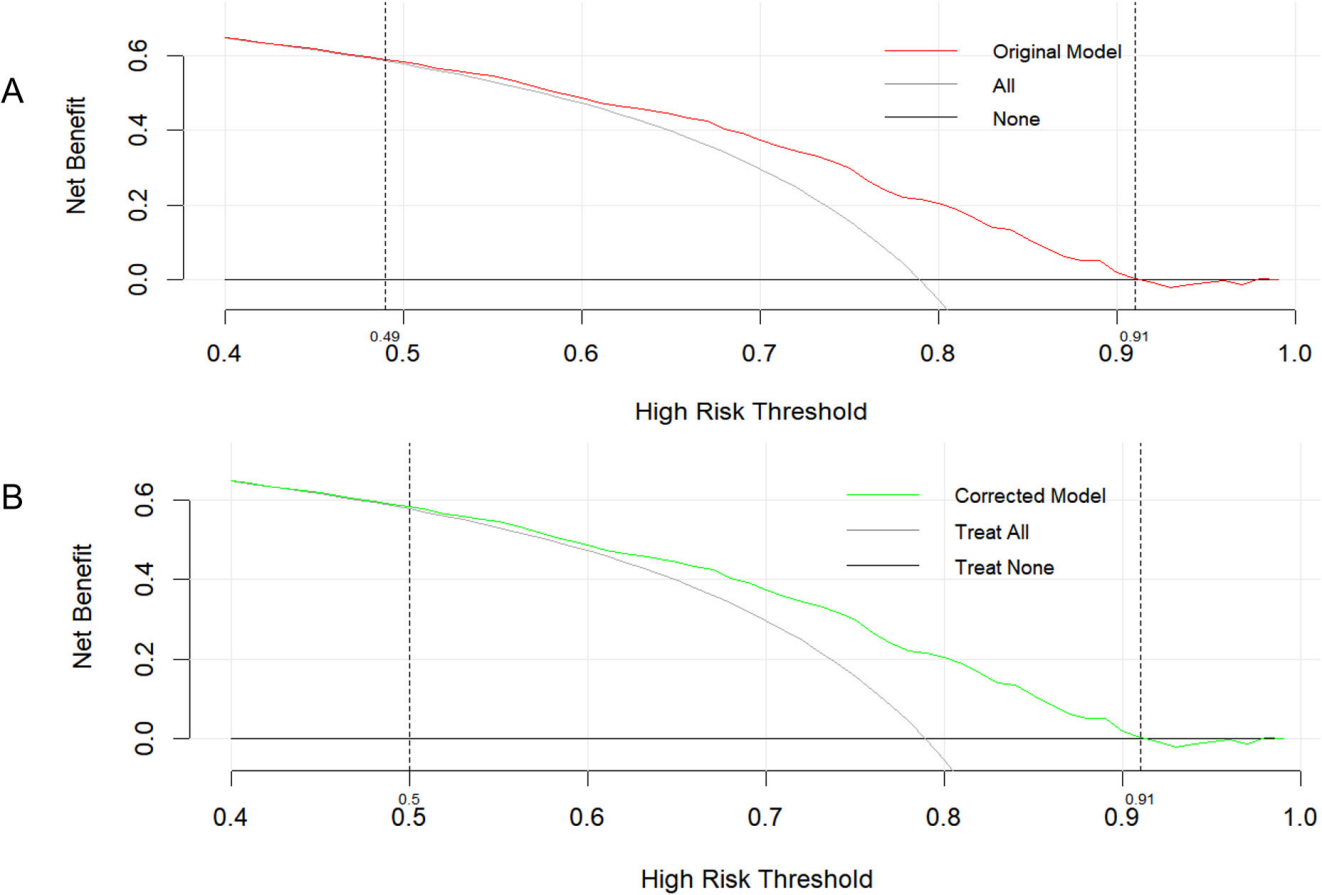
DCA of the original and corrected diagnostic model for obstructive CAD. **(A)** DCA of the original model. **(B)** DCA of the corrected diagnostic model. DCA, Decision curve analysis.

The DCA (**Figure 5**) demonstrated that the model provided a positive net clinical benefit across a wide range of threshold probabilities. In our highly selected, high-prevalence cohort, the specific clinical decision being modeled is the prioritization of patients for invasive CAG versus non-invasive testing or conservative medical therapy. Therefore, we focused our DCA narrative on high threshold probabilities. At a threshold probability of 0.8, the model yielded a net benefit of 0.205, which was higher than both the treat-all strategy (−0.055) and the treat-none strategy (0). This indicates that, in high-risk decision scenarios, the model could reduce unnecessary interventions while retaining a substantial proportion of true-positive cases (**Table 3**). The model demonstrated incremental clinical utility at high decision thresholds, suggesting its potential role in guiding the diagnosis or further referral for treatment of patients with suspected obstructive CAD. However, at an extreme threshold probability of 0.9, the net benefit decreased to 0.019, indicating diminished clinical applicability at extreme decision thresholds.

**Table 3:** Clinical net benefit of the obstructive CAD diagnostic model at selected thresholds.

Threshold	0.8	0.9
Model NB	0.205	0.019
Treat-all NB	-0.055	-1.109
Sensitivity (95% CI)	60.2% (57.4-62.8%)	18.3% (16.3-20.6%)
Specificity (95% CI)	68.0% (62.8-72.9%)	93.4% (90.2-95.8%)
PPV (95% CI)	87.6% (85.2-89.7%)	91.2% (87.1-94.3%)
NPV (95% CI)	31.3% (28.0-34.8%)	23.4% (21.2-25.7%)

NB, Net Benefit; PPV, Positive Predictive Value; NPV, Negative Predictive Value.

### 3.7 Sensitivity analysis in the age-matched cohort

To eliminate the confounding effect of age, we performed 1:1 PSM based on age and sex with a caliper width of 0.05. In the matched cohort, a multivariable logistic regression model including CHG, DELC, age, male sex, hypertension, and Scr was refitted to OR and 95% CI. After matching, 338 pairs of obstructive CAD and non-obstructive CAD patients (total N = 676) were successfully matched, with no significant difference in age between the two groups (58.3 years vs. 59.7 years, P = 0.091). In the multivariable analysis of the matched cohort (**Table 4**), the CHG index (OR = 9.901, 95% CI: 6.333–15.919, P < 0.001) and Scr remained independent predictors of obstructive CAD, whereas DELC and hypertension were no longer statistically significant (**Supplementary Figure S3**).

**Table 4:** Multivariable analysis in the PSM cohort.

Variable	Odds ratio (OR)	95% CI	P value
CHG	9.901	6.333 - 15.919	<0.001
DELC	0.891	0.606 -1.309	0.556
Age	0.987	0.969 - 1.006	0.169
Male	0.839	0.562 - 1.249	0.390
Hypertension	1.110	0.785 - 1.571	0.554
Scr	1.015	1.003 -1.028	0.023

Data derived from a 1:1 propensity score-matched sub-cohort comprising 338 patients with obstructive CAD and 338 non-obstructive controls. Age and sex were completely balanced across groups.

## 4 Discussion

This study investigated the diagnostic correlations of the CHG index and DELC with the presence of angiographic obstructive CAD. We found that among the 1,645 study subjects, both the proportion of DELC and CHG levels in the obstructive CAD group were significantly higher than those in the non-obstructive group. Furthermore, RCS analysis revealed that CHG levels were positively correlated with obstructive CAD risk (P < 0.001), exhibiting a linear relationship. The CHG index and DELC emerged as independent associated factors for obstructive CAD. By integrating them with traditional risk factors—namely male sex, age, hypertension, and Scr—we constructed a diagnostic model for angiographic obstructive CAD. Following bootstrap internal validation, the model demonstrated moderate discriminative ability, acceptable calibration, and potential clinical utility for specialized patient triage.

The pathological basis of obstructive CAD is the formation of AS, a process involving lipid infiltration, endothelial dysfunction, chronic inflammation, and oxidative stress (4, 5). In recent years, obstructive CAD has become a major global public health problem (1). Currently, CAG is the gold standard for diagnosing obstructive CAD. However, its application in inpatient triage is often limited by procedural complexity, substantial economic costs, and specific patient contraindications (14). Therefore, this study developed a simple, non-invasive diagnostic model to assist in inpatient triage and referral prioritization for hospitalized patients presenting with chest pain. Given that the study population consisted of hospitalized patients with suspected obstructive CAD based on the clinical judgment of physicians, the baseline prevalence was relatively high (approximately 79%), resulting in spectrum bias and selection bias. The predicted probabilities generated by this model are primarily applicable to similar high-risk clinical settings. Recalibration and external validation are explicitly required before extending its application to primary prevention screening or low-prevalence outpatient environments.

Both an increase in TC and a decrease in HDL-C are established risk factors for obstructive CAD (15). Elevated serum TC levels induce oxidative stress, damage endothelial cells, and trigger inflammatory responses (16). In contrast, high-density HDL-C exerts atheroprotective effects, primarily through the mechanism of reverse cholesterol transport, thereby reducing the risk of AS (17). Research has demonstrated that a low level of HDL-C is an independent risk factor for obstructive CAD (18). Elevated blood glucose levels can accelerate the progression of obstructive CAD by inducing oxidative stress and inflammatory responses (19). Furthermore, impaired glucose metabolism is closely linked to the initiation and progression of AS through shared pathophysiological pathways. Hyperglycemia promotes the accumulation of advanced glycation end products (AGEs), which exacerbate oxidative stress and inflammation through the activation of their receptor (20). Concurrently, hyperglycemia induces overproduction of superoxide, leading to endothelial dysfunction and insulin resistance (21). Moreover, oxidative stress drives the oxidative modification of LDL-C, a critical step in promoting foam cell formation and, consequently, the progression of AS (22). By integrating three key metabolic parameters directly influenced by endocrine regulation, the CHG index reflects the comprehensive state of lipid and glucose metabolism and effectively quantifies the cumulative burden of endocrine dysregulation on the cardiovascular system. In this study, our PCA identified the CHG index as the most significant contributor to the primary data variance (Dim1), exhibiting a stronger vector magnitude than its individual components. Moreover, after eliminating the confounding effect of age via PSM, the CHG index remained a potent and independent predictor of obstructive CAD (OR = 9.90, 95% CI: 6.33–15.92, P < 0.001). Although the composite CHG index did not achieve a statistically significant improvement in NRI or IDI compared to its individual components (TC, HDL-C, and FBG) entered separately, it effectively simplifies the model by integrating three metabolic parameters into a single unified measure. This parsimonious approach preserves discriminative ability while substantially reducing model complexity and the risk of overfitting.

DELC is defined as a diagonal skin fold extending from the tragus to the posterior auricular border (8). One prevailing hypothesis posits that DELC reflects underlying degenerative changes in the microvascular structure and resultant tissue hypoperfusion (9). Since its initial description by the American physician Sanders T. Frank in 1973, several studies have substantiated the DELC as an independent risk marker for cardiovascular disease (23, 24). In our primary multivariable analysis, the presence of DELC was initially identified as an associated factor for obstructive CAD. Our statistical analysis yielded VIF consistently below 2, confirming the absence of severe multicollinearity. However, biological specificity must be distinguished from statistical independence. A pertinent consideration is the natural biological correlation between the presence of DELC and advancing age (25). After adjusting for age using PSM, the independent association of DELC was attenuated (P = 0.556). Furthermore, the DeLong test demonstrated that incorporating DELC into a model already containing age and the robust CHG index provided only marginal, non-significant incremental discriminative value (AUC improvement P = 0.490). Collectively, these findings confirm that DELC is not an independent mechanistic driver of coronary atherosclerosis, but rather a macroscopic, non-specific phenotype of systemic cumulative microvascular aging that is closely tied to chronological age. Despite its limited incremental statistical value, DELC was intentionally retained in the final diagnostic model due to its completely non-invasive and cost-free nature, as well as its advantages in real-time identification and ease of operation.

Scr is a central biomarker for renal function, and its elevation can trigger pathophysiological alterations that facilitate coronary atherosclerosis (26, 27). Concurrently, age and hypertension, are established traditional risk factors for obstructive CAD (27). Based on the above, we constructed a diagnostic model visualized as a nomogram. By mapping each predictor to a point value, clinicians can intuitively derive the predicted probability of angiographic obstructive CAD. This approach provides clinicians in acute or specialized cardiovascular triage settings with a concise tool for clinical decision-making. Patients identified as relatively lower risk could be prioritized for non-invasive functional testing or optimized medical therapy, whereas those stratified as high risk can be expedited for confirmatory CAG to avoid management delays.

This study is subject to certain limitations. First, as a cross-sectional study derived from hospitalized patients undergoing CAG, the cohort exhibited a high prevalence of obstructive CAD. This inevitably introduced spectrum and selection biases, limiting the generalizability of our findings to lower-risk populations. Consequently, the model currently serves as an adjunctive risk assessment tool strictly for high-risk suspected patients. Second, although the DCA curve demonstrated diagnostic auxiliary value, the high disease prevalence likely led to an overestimation of the net clinical benefit. Third, single measurements of metabolic parameters (TC, HDL-C, FBG) may not completely capture an individual’s long-term profile due to biological variability. Fourth, due to the retrospective nature of the data, we were unable to document patients’ baseline medication history (including statins and antidiabetic drugs) or exact disease duration. Since these medications directly influence lipid and glucose levels, this may have partially masked true metabolic profiles, leading to residual confounding and potentially attenuating the diagnostic performance of the CHG index. Fifth, our complete-case analysis excluded patients with missing critical data. As data for the excluded patients are no longer available for direct comparison, it is plausible that they differed systematically from those retained, which may have introduced selection bias and affected the generalizability of our findings. Finally, given the cross-sectional design, this study cannot definitively prove a causal relationship between the CHG index and obstructive CAD risk.

## 5 Conclusion

This study revealed a positive linear association between the CHG index and the presence of angiographic obstructive CAD. Building on this, we developed and internally validated a comprehensive diagnostic model integrating the CHG index, DELC, male sex, age, hypertension, and Scr. This model exhibits moderate discriminative ability and potential utility for risk stratification in high-risk hospitalized patients, pending external validation in broader populations.

## Data Availability

The datasets used and evaluated in this study can be obtained from the corresponding author upon making a reasonable request. Requests to access the datasets should be directed to Donglei Luo, dongleiluocn@aliyun.com.
