# Supplementary material for: Diagnostic model for angiographic obstructive coronary artery disease combining CHG, DELC, and traditional risk factors: a bootstrap validation study
Source: Front Endocrinol (Lausanne). 2026 Apr 17;17:1799141. doi: 10.3389/fendo.2026.1799141 (PMC13133684; doi:10.3389/fendo.2026.1799141)
Supplement: Supplementary file 1 [file DataSheet1.docx]

Supplementary material


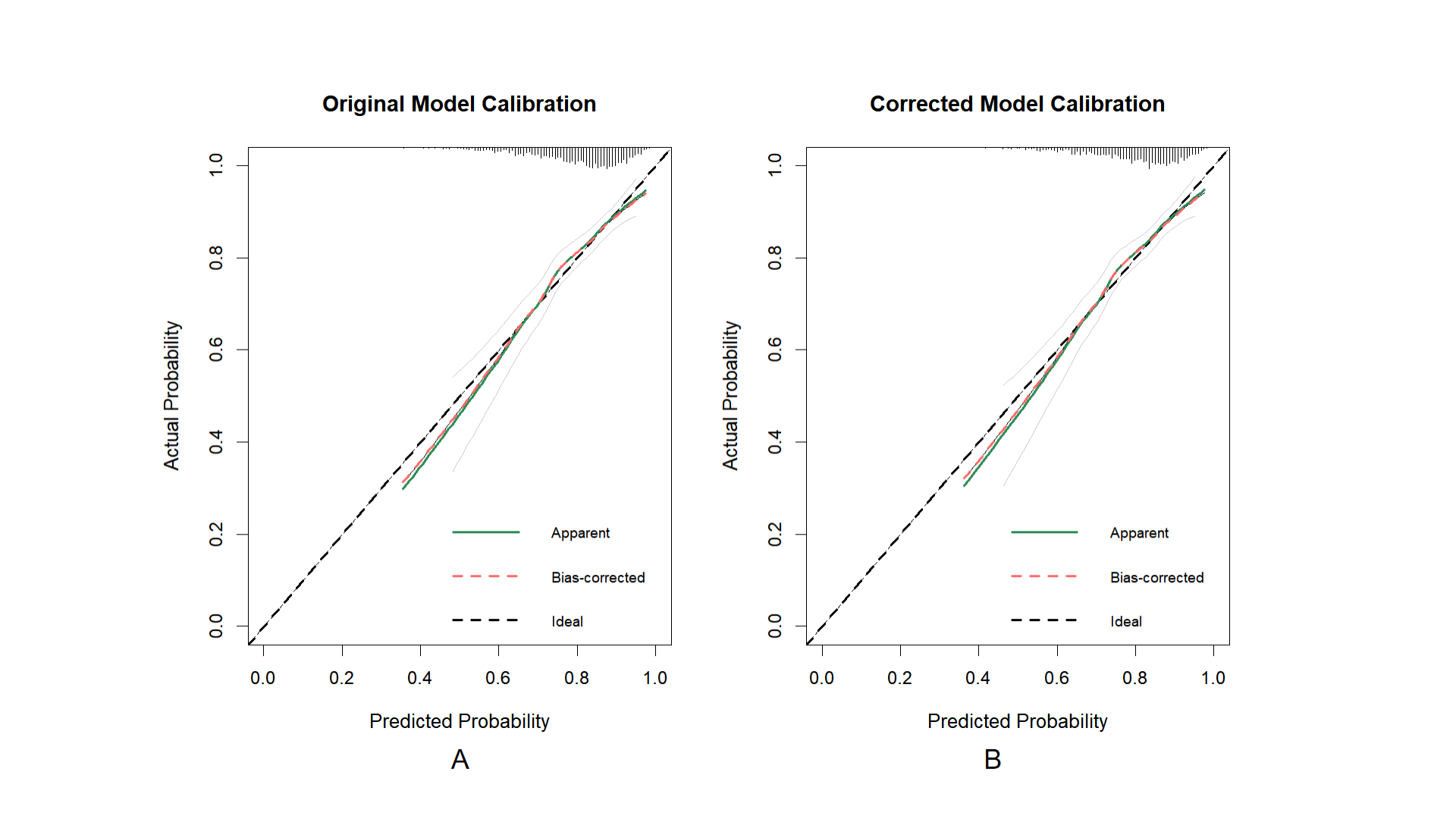


**Fig. S1** Calibration plots of the original and corrected diagnostic model for obstructive CAD. **A** Calibration plot based on the apparent predictions of the original model. **B** Calibration plot based on the bias-corrected predictions following internal validation via 500 bootstrap resamples.

Interpretive Description: The calibration curve evaluates the agreement between the nomogram-predicted probabilities of obstructive CAD (x-axis) and the actual observed event rates (y-axis). The dashed 45-degree line represents ideal, perfect calibration. In both the apparent and bias-corrected plots, the calibration curves (green solid line and red dashed line, respectively) closely adhere to the ideal diagonal line. This indicates that the diagnostic model possesses high absolute accuracy and is well-calibrated across a wide range of predicted risks within this high-prevalence cohort, without severe overestimation or underestimation.


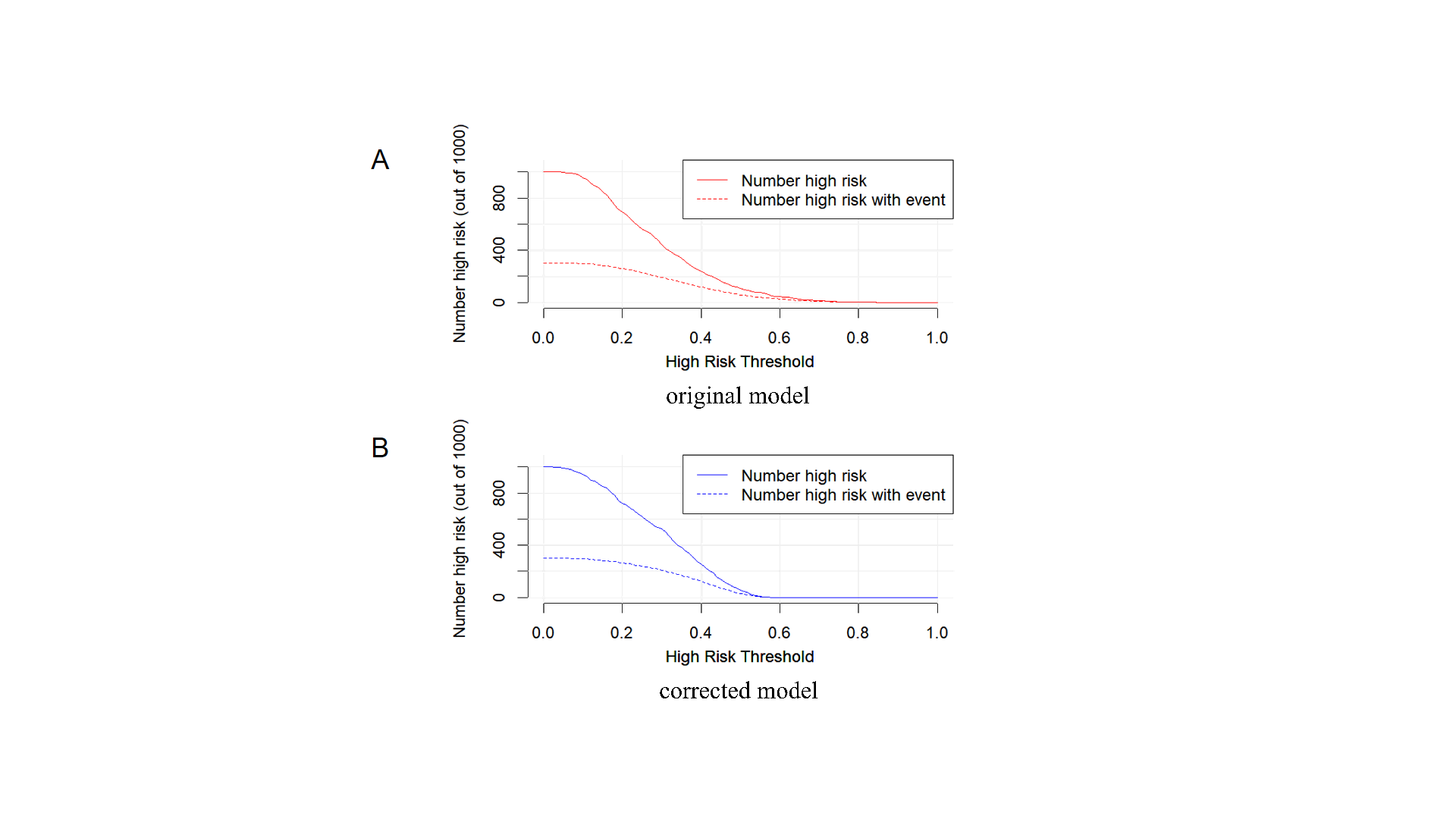


**Fig. S2** Clinical Impact Curve (CIC) of the original and corrected diagnostic model for obstructive CAD. **A** CIC of the original model based on apparent predictions. **B** CIC of the corrected model based on bias-corrected predictions following 500 bootstrap resamples.

Interpretive Description: In our clinical context of inpatient triage, the CIC visually estimates the clinical utility of the nomogram across a hypothetical population of 1,000 patients. The solid line (“Number high risk”) represents the total number of patients who would be classified as high risk at each threshold probability (x-axis) and therefore referred for invasive coronary angiography (CAG). The dashed line (“Number high risk with event”) represents the true-positive cases among those referred. The curves were generated based on the actual high baseline prevalence of obstructive CAD in our derivation cohort (approximately 79%). The close proximity between the solid and dashed lines at moderate-to-high threshold probabilities indicates that the vast majority of patients classified as high risk by the model truly have obstructive CAD. This confirms the model's substantial value in prioritizing high-risk patients for intervention while effectively minimizing unnecessary invasive procedures (false positives).
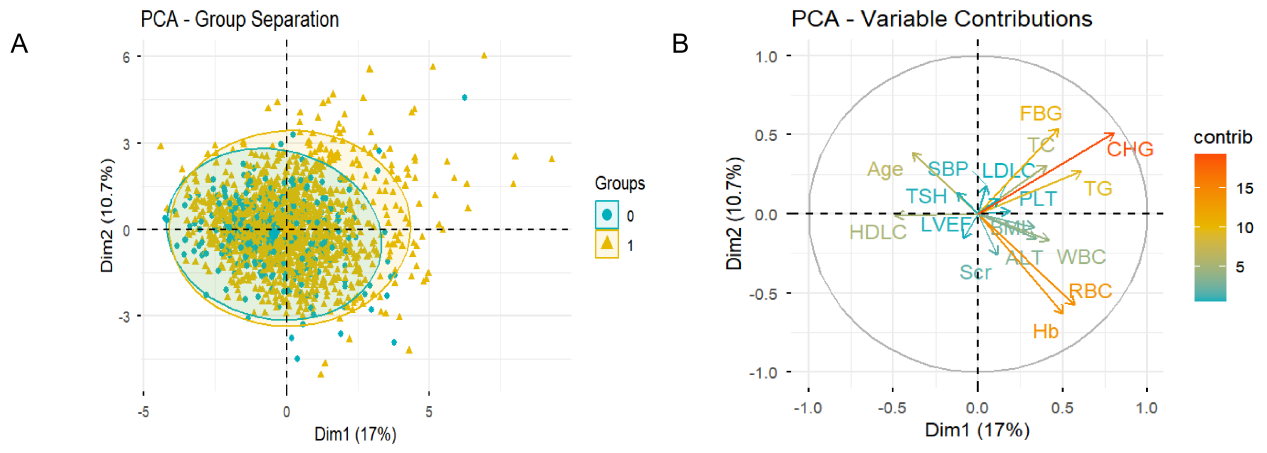


**Fig. S3**  Principal Component Analysis (PCA) group separation and variable contribution plots.

**A** PCA score plot of the study population. Green circles and yellow triangles represent patients without and with obstructive CAD, respectively.

Interpretive Description: The substantial multidimensional overlap between the two groups indicates that no clear, natural separation can be achieved using a simple linear combination of the selected baseline variables. This data complexity underscores the necessity of employing multivariable logistic regression modeling for accurate diagnostic discrimination.

**B** PCA variable contribution plot. The vectors represent the magnitude and direction of each continuous variable's contribution to the principal components.

Interpretive Description: The CHG index exhibited the highest contribution to the first principal component (Dim1), followed by hemoglobin (Hb) and red blood cell count (RBC) for Dim2. The dominant vector magnitude of the CHG index highlights its central role in characterizing the complex metabolic profile associated with obstructive CAD, supporting its integration as a core predictor in the final nomogram.
